# HbxB Is a Key Regulator for Stress Response and β-Glucan Biogenesis in *Aspergillus nidulans*

**DOI:** 10.3390/microorganisms9010144

**Published:** 2021-01-11

**Authors:** Sung-Hun Son, Mi-Kyung Lee, Ye-Eun Son, Hee-Soo Park

**Affiliations:** 1 School of Food Science and Biotechnology Kyungpook National University, Daegu 41566, Korea; rk05555@naver.com (S.-H.S.); thsdpdms0407@naver.com (Y.-E.S.); 2 Biological Resource Center (BRC), Korea Research Institute of Bioscience and Biotechnology (KRIBB), Jeongeup-si 56212, Korea; miklee1010@kribb.re.kr; 3 Department of Integrative Biology, Kyungpook National University, Daegu 41566, Korea

**Keywords:** conidia, homeobox domain, RNA-sequencing analysis, transcription factor, sterigmatocystin, *Aspergillus nidulans*

## Abstract

Homeobox transcription factors are conserved in eukaryotes and act as multi-functional transcription factors in filamentous fungi. Previously, it was demonstrated that HbxB governs fungal development and spore viability in *Aspergillus nidulans*. Here, the role of HbxB in *A. nidulans* was further characterized. RNA-sequencing revealed that HbxB affects the transcriptomic levels of genes associated with trehalose biosynthesis and response to thermal, oxidative, and radiation stresses in asexual spores called conidia. A phenotypic analysis found that *hbxB* deletion mutant conidia were more sensitive to ultraviolet stress. The loss of *hbxB* increased the mRNA expression of genes associated with β-glucan degradation and decreased the amount of β-glucan in conidia. In addition, *hbxB* deletion affected the expression of the sterigmatocystin gene cluster and the amount of sterigmatocystin. Overall, these results indicated that HbxB is a key transcription factor regulating trehalose biosynthesis, stress tolerance, β-glucan degradation, and sterigmatocystin production in *A.* *nidulans* conidia.

## 1. Introduction

Asexual spores are the main reproductive cells in most filamentous fungi [1,2]. Asexual spores are widespread in environmental niches, survive in harsh conditions, and germinate until appropriate conditions [1]. To survive in aggressive environmental conditions, asexual spores contain protective layers on their cell wall, unlike hyphae [3]. In addition, several signal pathways and regulators are involved in protecting from a myriad of environmental stresses [4,5].

The process of asexual spore production in filamentous fungi has been mainly studied in the model fungus *Aspergillus nidulans*, as various genetic and molecular techniques have been developed [6,7]. *A. nidulans* reproduces primarily through asexual development and produces an asexual-specific structure called conidiophore bearing long chains of asexual spores termed as conidia [1]. The process of conidiophore production is regulated by a variety of regulators, such as upstream regulators (FluG and FlbB-E), central regulators (BrlA, AbaA, and WetA), and feedback regulators (VosA and VelB) [8]. For conidia formation and maturation, three transcription factors—WetA, VosA, and VelB—mainly regulate the mRNA expression of spore-specific and developmental genes [9,10,11,12,13,14]. These regulators also coordinate the biosynthesis of trehalose, a key component for environmental stress tolerance, and β-glucan, a key polysaccharide for cell wall integrity [11,13,15]. With these transcription factors, several regulators, such as AtfA, VadA, and CatA, are involved in the process of spore tolerance against environmental stresses, maturation, dormancy, and germination [16,17,18,19,20].

Homeobox domain-containing proteins are found in animals, plants, yeast, and filamentous fungi [21,22]. These proteins contain homeobox DNA-binding motifs and control the transcription of a variety of genes [23,24]. Previous studies have demonstrated that homeobox proteins play diverse roles in fungal growth, differentiation, secondary metabolism, and pathogenesis in several Basidiomycota and Ascomycota [25,26,27]. For example, mating-type proteins encoded by *MAT***a** and *MAT*α form homocomplexes or heterocomplexes and regulate gene expression and yeast differentiation in the model yeast *Saccharomyces cerevisiae* [28]. In the pathogenic fungus *Candida albicans*, *GRF10* is involved in filamentous growth, biofilm formation, and virulence [29,30]. In *Aspergillus flavus*, Hbx1 plays an important role in fungal differentiation and secondary metabolism [31]. Transcriptomic and phenotypic analyses found that the deletion of *hbx1* affects the mRNA expression of developmental genes and secondary metabolite gene clusters [32]. In the human pathogenic fungus *Aspergillus fumigatus*, HbxA, a homolog of Hbx1, is a key regulator for asexual development, secondary metabolism, and pathogenesis [33].

A recent study indicated that the *A. nidulans* genome contains eight *hbx* genes [34]. Among these genes, *hbxA* and *hbxB* are essential for appropriate hyphal growth, conidiophore production, and cleistothecia formation in *A. nidulans*. In addition, *hbxB* deletion leads to decreased amounts of trehalose, conidia viability, and thermal tolerance in conidia, implying that HbxB plays a key role in conidia maturation. To further test the role of HbxB in conidia, we conducted transcriptomic and phenotypic analyses in this study.

## 2. Materials and Methods 

### 2.1. Strains and Media

In this study, control (THS30, *pyrG89*; *AfupyrG*^+^) [11], *hbxB* deletion mutant (Δ*hbxB*, TSH1, *pyrG89*; *pyroA4*; Δ*hbxB*::*AfupyrG*^+^), and *hbxB*-complemented (C’ *hbxB*, TSH7, *pyrG89*; *pyroA*::*hbxB*(p)::*hbxB*::FLAG_3x_::*pyroA*; Δ*hbxB*::*AfupyrG*^+^) [34] strains were used. These fungal strains were grown on minimal medium with 1% glucose (MMG) for general purpose [35].

### 2.2. RNA-Sequencing (RNA-Seq) Analysis

The whole processes of RNA-seq analysis were performed as described previously [20]. The Δ*hbxB* conidia were collected from the plates after 2 days of culture and filtrated through Miracloth (Calbiochem, San Diego, CA, USA). The total RNA from conidia was extracted using Trizol reagent (Invitrogen, Carlsbad, CA, USA). After RNA extraction, DNase I (Promega, Madison, WI, USA) was used for the removal of DNA contamination from RNA samples and then further purification using the RNeasy Mini Kit (Qiagen, Germantown, MD, USA).

Complementary DNA (cDNA) library preparation and RNA-seq were performed by Theragen Bio Co., Ltd. (Seongnam, South Korea). Briefly, mRNA from total RNA was isolated from magnetic beads with oligo(dT). The cDNA library for RNA-seq was prepared using the TruSeq Stranded mRNA Sample Prep Kit (Illumina, San Diego, CA, USA). The library was evaluated using the Agilent High Sensitivity DNA Kit (Agilent Technologies, Santa Clara, CA, USA) and sequenced using an Illumina HiSeq2500 sequencer (Illumina). All RNA-seq data files are available from the National Center for Biotechnology Information BioProject database (PRJNA681980).

RNA-seq data were analyzed as reported previously [20]. Briefly, the filtered readings were mapped onto the *A. nidulans* A4 transcriptome [36] using the aligner STAR version 2.3.0e software [37]. Gene expression levels were measured using Cufflinks version 2.1.1 [38]. Gene-level count data were generated using the HTSeq-count version 0.5.4p3 tool [39] with the options “-m intersection-nonempty” and “-r option considering paired-end sequence.” Differentially expressed genes (DEGs) were identified using the R package TCC [40] on the basis of the read count data. Normalization factors were calculated using the iterative DEGES/edgeR method. DEGs were identified on the basis of a q-value threshold of less than 0.05.

### 2.3. Gene Ontology (GO) Term Enrichment Analysis

GO term enrichment analysis was shown using the Gene Ontology Slim Mapper at AspGD [36]. A GO-based trend test was conducted using Fisher’s exact test. *p* < 0.001 was considered statistically significant to identify the significant category from the analyzed DEGs.

### 2.4. Quantitative Reverse Transcription Polymerase Chain Reaction (qRT-PCR) Analysis

For qRT-PCR analysis, total RNAs from control, Δ*hbxB*, and C’ *hbxB* conidia were extracted using the method mentioned above. The GoScript Reverse Transcription System (Promega) was used for cDNA synthesis. The iTaq Universal SYBR Green Supermix and the CFX96 Touch Real-Time PCR Detection System (both from Bio-Rad, Hercules, CA, USA) were used for qRT-PCR. The 2^−ΔΔCT^ method was used for calculating the expression levels of the target genes, and β-actin was used as an endogenous control. The gene-specific primers used in this study are listed in Table S1. This assay was carried out in triplicate.

### 2.5. Ultraviolet (UV) Stress Tolerance Assay 

The UV stress tolerance assay was carried out as described previously [41]. Briefly, about 100 conidia were spread on MMG plates and irradiated using the UV Spectrolinke XL-1000 UV crosslinker (Thomas Scientific, Swedesboro, NJ, USA). After irradiation, the plates were incubated at 37 °C for 48 h, and the colony numbers were counted. The survival rate was calculated by comparing the number of colonies in the UV-treated plate and the untreated plate.

### 2.6. β-. Glucan Assay

The amounts of β-1,3-glucan in conidia were measured by the Glucatell assay (Associates of Cape Cod, East Falmouth, MA, USA) as described previously [11,42]. Briefly, 2-day-old conidia from control and mutant strains were collected using double-distilled water. Conidia suspension was mixed with Glucatell reagent and incubated at 37 °C for 30 min. After incubation, diazo-reagents were added to stop the reaction, and the optical density was determined at 540 nm.

### 2.7. Sterigmatocystin Extraction and Thin-Layer Chromatography (TLC) Analysis

The extraction of sterigmatocystin from 2-day-old conidia was conducted as described previously [20,43]. About 10^9^ conidia were mixed with CHCl_3_ and 0.5 mm zirconia/silica beads (RPI, Mt. Prospect, IL, USA) and disrupted using a Mini-Beadbeater (BioSpec Products, Inc., Bartlesville, OK, USA). After centrifugation, the organic phase was transferred to new vials and evaporated. Each sample was resuspended in CHCl_3_, spotted onto a TLC silica plate (Kiesel gel 60, 0.25 mm; Merck, Kenilworth, NJ, USA), and resolved in toluene/ethyl acetate/acetic acid (8:1:1, v/v). The TLC plates were treated with 1% aluminum hydroxide hydrate (Sigma, St. Louis, MO, USA). The images of the TLC plates were captured after UV exposure (366 nm). The spot intensities of sterigmatocystin were quantified using ImageJ software.

### 2.8. Statistical Analysis

The statistical differences between control and Δ*hbxB* strains were evaluated by Student’s unpaired *t*-test. The mean ± standard deviation are shown. *p* < 0.05 was considered significant.

## 3. Results

### 3.1. Regulatory Role of HbxB in Conidia

A previous study demonstrated that HbxB governs conidial viability, conidial trehalose biosynthesis, and stress response in conidia [34]. On the basis of these results, we hypothesized that HbxB is a transcription factor that can regulate the mRNA expression of a variety of genes in conidia. To test this hypothesis, RNA-seq analysis using control and Δ*hbxB* mutant conidia were conducted. The transcriptomic analysis results found that a total of 6230 genes were differentially expressed between control and Δ*hbxB* mutant conidia (fold change > 2.0; q < 0.05; Figure S1). The mRNA levels of 3202 genes were upregulated, and the transcripts of 3028 genes were downregulated in Δ*hbxB* mutant conidia compared to control strain.

**Figure 1:**
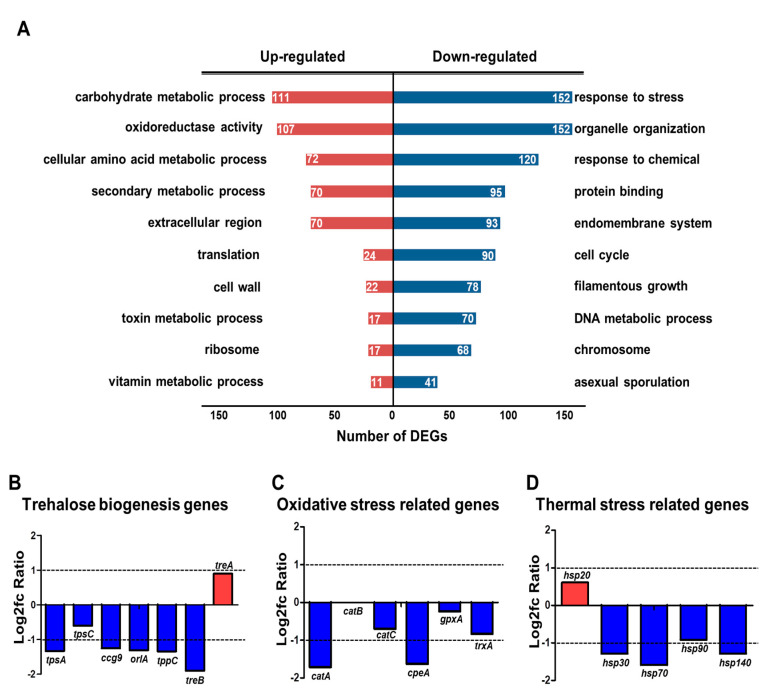
Transcriptomic analysis in *hbxB* deletion mutant conidia. (**A**) Gene ontology (GO) term enrichment analysis of upregulated and downregulated genes in *hbxB* deletion mutant conidia. (**B**–**D**) mRNA expression of genes associated with trehalose biogenesis (**B**), oxidative stress response (**C**), and thermal stress response (**D**) in *hbxB* deletion mutant conidia.

To further elucidate the regulatory role of HbxB, we performed GO functional enrichment analysis using RNA-seq results and the ASPGD platform [36]. GO analysis revealed that up-regulated DEGs were enriched in mainly “carbohydrate metabolic process,” “oxidoreductase activity,” “cellular amino acid metabolic process,” “secondary metabolic process,” “translation,” and “cell wall” (Figure 1A). The downregulated genes were mainly associated with “response to stress,” “organelle organization,” “response to chemical,” “protein binding,” and “endomembrane system” (Figure 1A).

A previous study reported that hbxB deletion decreases the trehalose content and stress tolerance in conidia [34], suggesting that the mRNA expression of the related genes can also be affected. Therefore, the mRNA levels of genes associated with trehalose biosynthesis and response to oxidative and thermal stresses were evaluated. As shown in Figure 1B, the expression of *tpsA*, *orlA*, and *tpsC*, which are involved in trehalose biosynthesis [44], was decreased. In addition, the transcript levels of genes associated with oxidative and thermal stress response were decreased (Figure 1C). The RNA-seq results were verified by qRT-PCR analysis (Figure S2). Overall, these transcriptomic results supported the reason why and how *hbxB* deletion affects conidial phenotypes.

### 3.2. Function of HbxB in UV Stress Response

**Figure 2:**
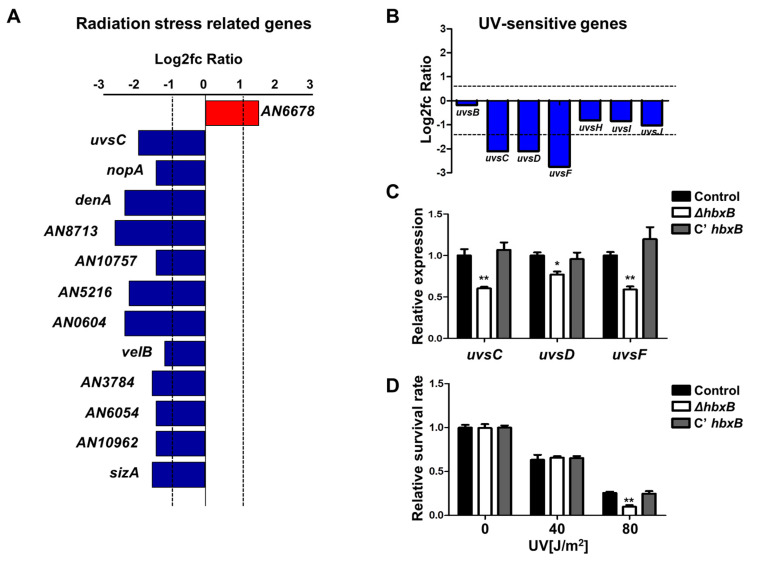
Role of *hbxB* on UV stress tolerance in *Aspergillus nidulans* conidia. (**A**–**C**) mRNA expression of genes associated with radiation stress gene clusters in *hbxB* deletion mutant conidia (*p* < 0.05, log2fc > 1.0). (**B**) mRNA levels of genes involved in UV-sensitive gene clusters in *hbxB* deletion mutant conidia. (**C**) mRNA expression of *uvsC*, *uvsD*, and *uvsF* in control (TNJ36), Δ*hbxB* (TSH1.1), and C’ *hbxB* (TSH7.1) strain conidia were verified by qRT-PCR analysis. * *p* < 0.05; ** *p* < 0.01, differences between control and Δ*hbxB* conidia. (**D**) UV sensitivity of control (TNJ36), Δ*hbxB* (TSH1.1), and C’ *hbxB* (TSH7.1) strain conidia. ** *p* < 0.01, differences between control and Δ*hbxB* conidia.

As shown in Figure 1A, many genes associated with stress response were downregulated in *hbxB* mutant conidia. The list of these genes was screened, and several genes associated with radiation and UV stress response were downregulated (Figure 2A–C), suggesting that *hbxB* deletion can also affect the UV stress tolerance. To confirm this, we irradiated conidia of control, Δ*hbxB*, and C’ *hbxB* strains using a UV crosslinker. As shown in Figure 2D, the resistance of Δ*hbxB* conidia to UV stress was less than those of control and C’ *hbxB* conidia. Taken together, these results demonstrated that HbxB is required for an appropriate response to UV and other environmental stresses.

### 3.3. Function of HbxB in UV Stress Response 

**Figure 3:**
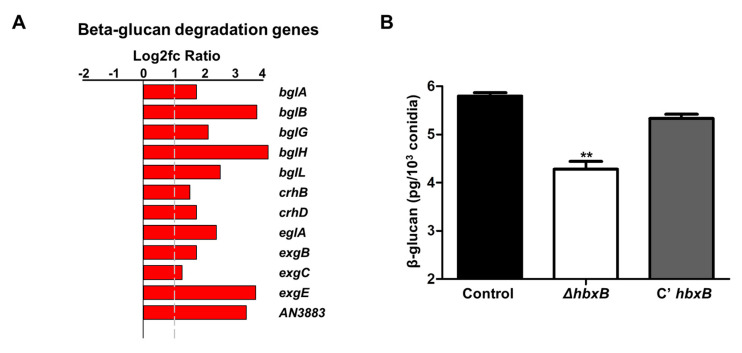
Role of *hbxB* on β-glucan degradation in *A. nidulans* conidia. (**A**) mRNA expression of genes associated with β-glucan degradation in *hbxB* deletion mutant conidia. (**B**) Amount of β-glucan in control (TNJ36), Δ*hbxB* (TSH1.1), and C’ *hbxB* (TSH7.1) strain conidia. ** *p* < 0.01, differences between control and Δ*hbxB* conidia.

RNA-seq analysis results showed that the genes involved in cell wall integrity were also affected by *hbxB* deletion (Figure 1A). Among them, 12 genes associated with β-glucan degradation were upregulated in Δ*hbxB* conidia (Table 1; Figure 3A). To test the phenotypic change according to the alteration of gene expression, we examined the amount of β-glucan in conidia. As shown in Figure 3B, β-glucan production was decreased in the Δ*hbxB* mutant conidia compared to control and C’ *hbxB* conidia. These results demonstrated that HbxB affects the production of β-glucan in conidia by regulating the mRNA expression of β-glucan degradation-related genes

### 3.4. HbxB Affects Sterigmatocystin Production in Conidia.

**Figure 4:**
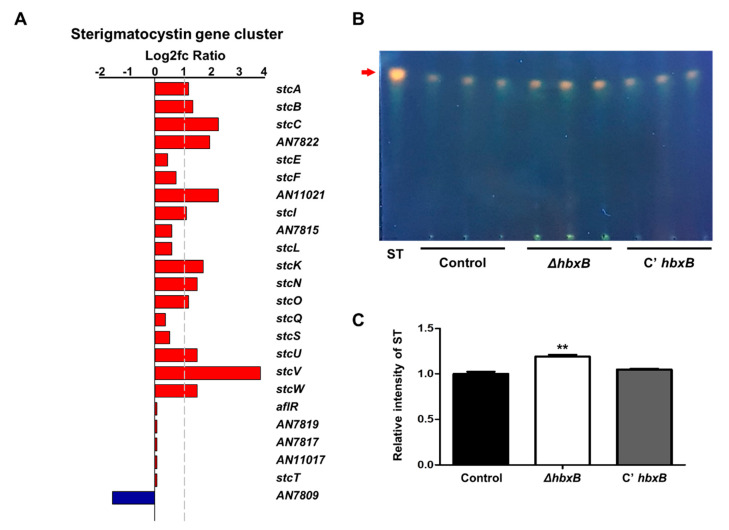
Role of *hbxB* for sterigmatocystin production in *A. nidulans* conidia. (**A**) mRNA expression of genes associated with the sterigmatocystin gene in *hbxB* deletion mutant conidia was verified by RNA-sequencing (RNA-seq) analysis. (**B**) Thin-layer chromatography (TLC) images of sterigmatocystin from control (TNJ36), Δ*hbxB* (TSH1.1), and C’ *hbxB* (TSH7.1) strain conidia. (**C**) Relative intensity of sterigmatocystin in (**B**) using ImageJ software. ** *p* < 0.01, differences between control and Δ*hbxB* conidia.

As mentioned above, *hbxB* deletion affects gene expression for secondary metabolic processes (Figure 1A). Among the secondary metabolite gene clusters, the mRNA expression of several genes involved in the sterigmatocystin gene cluster was upregulated in the Δ*hbxB* mutant conidia compared to control and C’ *hbxB* conidia (Figure 4A). In addition, Δ*hbxB* conidia had a higher amount of sterigmatocystin than control and C’ *hbxB* conidia (Figure 4B,C). These results suggested that HbxB is essential for the proper production of sterigmatocystin in conidia.

## 4. Discussion

Homeobox proteins are conserved in most filamentous fungi and play diverse roles in fungal development and metabolisms [21,26]. Most *Aspergillus* species contain eight homeobox proteins, of which HbxA (or Hbx1) has been mainly studied [31,33]. In three *Aspergillus* species, including *Aspergillus nidulans*, *Aspergillus fumigatus*, and *Aspergillus flavus*, *hbxA* (or *hbx1*) deletion affects hyphal growth, conidiophore formation, and secondary metabolite production. The roles of *hbxA* in asexual development has been demonstrated in other fungi, such as *Fusarium graminearum*, *Magnaporthe oryzae*, and *Ustilaginoidea virens*, suggesting that the roles of HbxA (or HbxA orthologs) are conserved in fungal development [25,27,45]. Unlike HbxA, the function of other homeobox domain-containing proteins including HbxB (or HbxB orthologs) has not been studied well in other fungi. Recently, our study revealed the role of HbxB in *A. nidulans* [34]. HbxB acts as a key regulator for the balance between asexual and sexual development in *A. nidulans*. In this study, we first reported the function of HbxB in spores through the transcriptomic and phenotypic analyses. These results can provide an insight into the basic knowledge about the function of the HbxB orthologs in other fungal species. 

One of the important findings in this study is that HbxB is important for response to various stresses in conidia. RNA-seq results found that the mRNA expression of approximately 150 genes associated with stress response was downregulated in Δ*hbxB* conidia (Figure 1). These are associated with response to oxidative (*catA*, *cpeA*, and *trxA*), thermal (*hsp30*, *hsp70*, and *hsp140*), radiation (*nopA*, *denA*, *velB*, and *sizA*), and UV (*uvsC*, *uvsD*, and *uvsF*) stresses (Figure 1 and Figure 2). This decreased mRNA expression of stress-related genes might affect the phenotype and increase susceptibility to various stresses, including thermal, oxidative, and UV stresses. In addition, decreased mRNA expression of trehalose biosynthesis genes and the amount of trehalose in Δ*hbxB* conidia can also affect the response to various stresses. These results supported the idea that HbxB is a key controller for the various stress responses in conidia. Although we found that HbxB affects mRNA expression of stress-related genes, the detailed molecular mechanism of HbxB has not been studied yet. Moreover, the genetic relationship between HbxB and other regulators involved in conidial stress response has not been studied. In conidia, the high-osmolarity glycerol (HOG) pathway and the velvet proteins control conidial stress tolerance in *A. nidulans* [4,46]. We can speculate that HbxB cross-talk with the HOG pathway or the velvet regulators for regulating mRNA expression of genes involved in conidial stress response. Further research will be needed to illuminate how conidial stress response is precisely regulated by these regulators.

Another finding in this study is that HbxB is involved in gene expression related to secondary metabolite gene clusters (Figure 4). In particular, HbxB can function as a negative regulator for sterigmatocystin production. The mRNA levels of several sterigmatocystin biosynthesis genes and the amount of sterigmatocystin were increased in Δ*hbxB* conidia. However, this result is the opposite of a previous result. In dark conditions for sexual development, *hbxB* deletion decreased sterigmatocystin production and the mRNA levels of *aflR*, encoding an activator of the sterigmatocystin gene cluster [34]. It was speculated that the function of HbxB works differently depending on the temporal or cell type-specific regulation, and additional studies are needed to reveal this.

**Figure 5:**
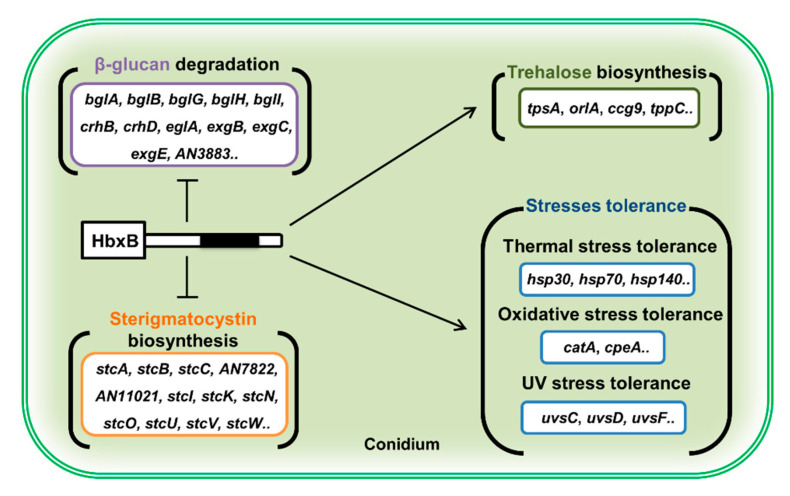
Proposed model depicting the role of HbxB in conidium (see the Discussion section).

Overall, this study suggests that HbxB has a multi-functional role in fungal development and metabolism in *A. nidulans*. During the developmental process, HbxB regulates the balance between asexual and sexual development. In conidia, HbxB regulates the mRNA levels of genes associated with stress response, β-glucan biosynthesis, trehalose biosynthesis, and secondary metabolism, thereby governing conidial stress response, primary and secondary metabolism, and conidial maturation (Figure 5). Although HbxB has been found to affect the transcription of thousands of genes, the direct targets of HbxB and the detailed molecular mechanism of HbxB are still unknown. It should be required for understanding conidiogenesis in *A. nidulans*.

## Figures and Tables

**Table 1:** Differentially expressed genes (DEGs) associated with cell wall integrity in Δ*hbxB* conidia.

Category (Number of Genes in the Category)	Upregulated Genes in Δ*hbxB* Conidia	Downregulated Genes in Δ*hbxB* Conidia
β-Glucan biosynthesis (12)	*gelB, crhD, sunA*	*gelD*
β-Glucan degradation (41)	*bglA, bglB, bglG, bglH, bglL, crhB, crhD, eglA, exgB, exgC, exgE, AN3883*	-
Chitin biosynthesis (17)	*chsF*	*chs7*
Chitin degradation (23)	*chiC, AN0221, AN0299*	*nagA, AN12280, AN8999*

## Data Availability

All RNA-seq data files are available from the National Center for Biotechnology Infor-mation BioProject database (PRJNA681980).

## Supplementary Materials

The following are available online at https://www.mdpi.com/2076-2607/9/1/144/s1: Figure S1. Heat map images of DEGs between control and Δ*hbxB* conidia (fold change > 2.0; *q* < 0.05). Figure S2. Levels of genes involved in trehalose biosynthesis (**A**) and oxidative stress response (**B**) were verified by qRT-PCR analysis. * *p* < 0.05; ** *p* < 0.01; *** *p* < 0.001, differences between control and Δ*hbxB* conidia. Table S1. Oligonucleotides used in this study.
