# Supplementary material for: HbxB Is a Key Regulator for Stress Response and β-Glucan Biogenesis in Aspergillus nidulans
Source: Microorganisms. 2021 Jan 11;9(1):144. doi: 10.3390/microorganisms9010144 (PMC7827800; doi:10.3390/microorganisms9010144)
Supplement: Supplementary file 1 [file microorganisms-09-00144-s001.pdf]

Figure S1

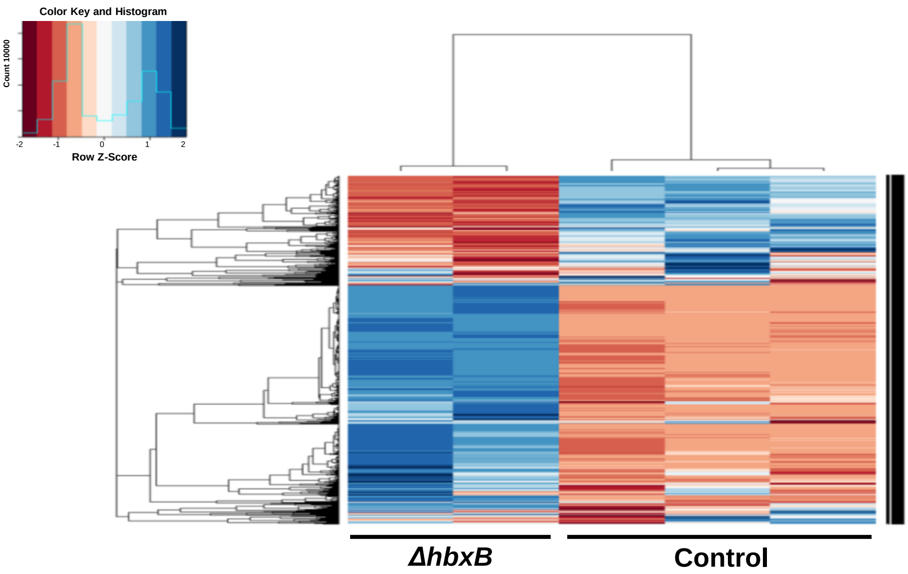

Total : 6,230 genes
(*p*-value < 0.05 and fold change > 1.0)

Figure S2

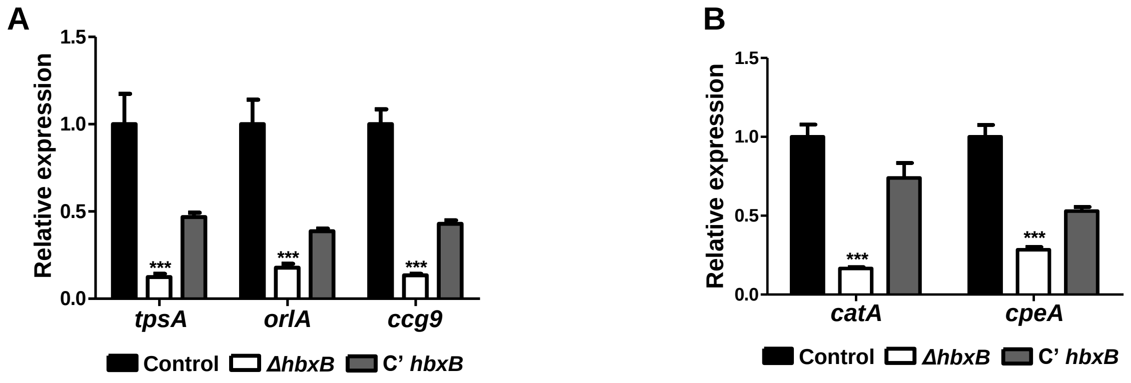
A
Relative expression
1.5
1.0
0.5
0.0
***
tpsA
orlA
ccg9
Control
ΔhbxB
C' hbxB
B
Relative expression
1.5
1.0
0.5
0.0
***
catA
cpeA
Control
ΔhbxB
C' hbxB

Table S1. Oligonucleotides used in this study.

Name	Sequence (5′ → 3′)	Purpose
**OHS0576**	GGTTGAAGTCGTCGGTTGAG	tpsA (AN5523)_RT_F
**OHS0577**	TGGAAACCGATGAGGTCACA	tpsA (AN5523)_RT_R
**OHS0616**	CTCCTACTCGCGTCACTTCT	orlA (AN3441)_RT_F
**OHS0617**	AGGAAAGACATCCACAGCCA	orlA (AN3441)_RT_R
**OHS1119**	GATTATTCGGCCCAGAGGGA	ccg9 (AN5021)_RT_F
**OHS1120**	ATGGCTTTCCACGTATTGGC	ccg9 (AN5021)_RT_R
**OHS1540**	TCGACTCAGCTACCTCCCTA	uvsC (AN1237)_RT_F
**OHS1541**	ACTGCAATGCCGAACTCATC	uvsC (AN1237)_RT_R
**OHS1536**	GACACCAAAGAAGCCCAAGG	uvsD (AN5165)_RT_F
**OHS1537**	TGGCTTGTCCGTCTTCTCTT	uvsD (AN5165)_RT_R
**OHS1538**	CGATGACCAACTCTGGACCT	uvsF (AN6303)_RT_F
**OHS1539**	TAAAGTTCGCCTTTGCGCTT	uvsF (AN6303)_RT_R
**OHS0599**	GCGCGAAGAAGACTTCAAC	aflR (AN7820)_ RT_F
**OHS0600**	TGCAATAACTGCCGACGAC	aflR (AN7820)_ RT_R
**OHS0946**	GGATCTGCCAAAGCGAACAT	stcA (AN7825)_ RT_F
**OHS0947**	CCACAGTGAGGAGGAATGGT	stcA (AN7825)_ RT_R
**OHS0604**	GCTACTGTTCCAGGCGACTA	stcE (AN7821)_ RT_F
**OHS0605**	CACAGCTCTCCATCTCGGTA	stcE (AN7821)_ RT_R
**OHS0602**	CGCATCATCCTCACAAGTTC	stcU (AN7806)_ RT_F
**OHS0603**	TGACCGTGATCTTCTTGTCG	stcU (AN7806)_ RT_R
